# Editorial: Advances in the diagnosis and prevention of diabetic neuropathy

**DOI:** 10.3389/fendo.2022.1110928

**Published:** 2022-12-19

**Authors:** Charumathi Sabanayagam

**Affiliations:** ^1^ Ocular Epidemiology Research Group, Singapore Eye Research Institute, Singapore, Singapore; ^2^ The Ophthalmology and Visual Sciences Academic Clinical Program (EYE ACP), Duke-NUS Medical School, Singapore, Singapore

**Keywords:** Diabetic peripheral neuropathy, diagnosis, risk factor, biomarkers, recent advances

Diabetic Neuropathy (DN) is a common complication of type 1 and type 2 diabetes. Diabetic peripheral neuropathy (DPN) and cardiac autonomic neuropathy (CAN) lead to devastating complications like foot ulcers, lower limb amputations, major cardiovascular events, and sudden deaths (1). It is estimated that up to 50% of people with diabetes may be affected by some type of neuropathy making it a significant cause of disability and reduction in quality of life (2). Complications related to both DPN, and CAN are preventable if detected early and risk factors are optimally controlled through multidisciplinary care. However, due to the asymptomatic early stages of the disease progression, diagnosis is often a challenge. The issues are compounded by the threat of progression of disease to an irreversible stage if detection is delayed. This Research Topic collection aims to address these challenges by featuring and discussing the latest advances in diagnostic approaches to DN. In this collection, we have compiled 14 research articles from those submitted to the call for papers on “*Advances in the Diagnosis and Prevention of Diabetic Neuropathy*” topic and those received through regular submission route. The final collection of articles includes topics that summarize and evaluate the latest technologies in screening, diagnosis, and prediction of DPN.

Article type wise, the 14 research articles published under this collection include 2 Narrative Review, 1 Systematic Review and Meta-analysis, 1 Case Report, and 10 Original Research articles. These articles encompass four key areas related to recent advances in DPN:

1. Screening and diagnostic tests
2. Risk factors associated with DPN and prediction models for early detection of DPN
3. Potential biomarkers for diagnosing DPN
4. Novel techniques exploring mechanisms underlying DPN

## Screening and diagnostic tests

A narrative review by Yu gives an overview of the current screening technologies for detecting early DPN and summarises their advantages, disadvantages, and potential clinical application. The technologies assessed include quantitative sensory measurement, neurological function scoring system, corneal confocal microscopy (CCM), and high-frequency ultrasound. Carmichael et al. provide a comprehensive review of current knowledge and optimal approaches for screening and diagnosing DPN. Authors highlighted the limitations of the monofilament test, a commonly used screening test in primary care in terms of its inability to detect early-stage disease and high variability in sensitivity ranging from 43-93% compared to the gold standard, nerve conduction studies (NCS) for detecting late-stage DPN. They also assessed the performance of screening and diagnostic test currently available for early detection of DPN including the potential for several novel point of care devices (POCDs) such as Neuropad, DPNCheck and Sudoscan. Authors further highlighted the potential for CCM, a rapid non-invasive clinical assessment of corneal nerves in detecting small fibre neuropathy, the earliest manifestation of DPN and to assess its severity. Authors suggested that advancements in automated analysis softwares may improve clinical utility by overcoming the difficulty in manual analysis and technical expertise to quantify nerve pathology. Carmichael et al. also reported the results of a feasibility study using CCM to screen for DPN in patients with type 1 or type 2 diabetes attending primary care optometry settings in UK. Their study findings supported the current literature that CCM is a sensitive surrogate biomarker for DPN.

## Risk factors

Christensen et al. evaluated the association of glycemic variability (GV) assessed by means of continuous glucose monitoring (CGM) generated parameters and showed that GV was not associated with neither DPN nor CAN in a cohort of 133 young Danish adults with type 1 diabetes. Al-Saoudi et al. conducted a cross-sectional study assessing the association of advanced glycation end-products (AGEs) with CAN and distal symmetric polyneuropathy (DSPN) in 151 young adults with type 1 diabetes in Denmark. They concluded that higher levels of AGEs were associated with several measures of CAN and DSPN through diverse metabolic pathways including glycolytic dysfunction, lipid peroxidation and glucotoxicity. Liu et al. conducted a study comparing the prevalence and risk factors associated with DPN, peripheral artery disease (PAD) and foot deformity which are the common causes of diabetic foot using a large cohort of 3898 patients with diabetes from 11 hospitals in Beijing, China. They found the prevalence of foot deformities including callus to be higher (29.7%), followed by DPN (23.5%) while the prevalence of PAD to be lower with 11.6%. They found risk factors including higher systolic blood pressure, underweight, poor glycemic control, longer duration of diabetes, chronic kidney disease and cerebrovascular disease were commonly associated with 2 or more of the three conditions. Wang et al. developed and validated a nomogram model for predicting those at higher risk of developing diabetic foot using data collected from 1950 patients with type 2 diabetes attending a university hospital in China. The model based on traditional risk factors such as age, HbA1c, total and low-density lipoprotein cholesterol, smoking and drinking had good accuracy with area under the receive operating characteristic curve (AUC) of 0.857 in internal validation.

## Novel biomarkers

Three clinic-based studies reported cross-sectional association between novel biomarkers and DPN. Sun et al. reported higher levels of serum adiponectin to be positively associated with DPN independent of potential confounders in 219 Chinese type 2 diabetic patients aged 40-79 years. Jende et al. reported higher levels of troponin T (hsTNT) to be negatively correlated with markers of magnetic resonance peripheral nerve perfusion in a small case-control sample with and without diabetes suggesting that hsTNT may serve as a potential marker for assessing nerve perfusion in future studies of DN. Zhuang et al. demonstrated that plasma increased plasma levels of D-dimer to be independently associated with DPN in 393 patients with type 2 diabetes. Dong et al. performed a systematic review and meta-analysis of six studies that evaluated the performance of shear wave elastography (SWE) for tibial nerve stiffness as a quantitative biomarker complementary to neuroelectrophysiological examination for diagnosing DPN. They reported that SWE had good diagnostic accuracy for detecting DPN with summary sensitivity, and specificity of 75%, and 86% and area under the receiver operating characteristic curve (AUROC) of 0.86.

## Mechanistic studies

Chen et al. conducted a diffusion tensor imaging study to evaluate the differences in central neural mechanisms underlying erectile dysfunction due to type 2 diabetes (DM-ED) vs. psychological erectile dysfunction (pED) using the method of network-based statistic. Authors demonstrated differences in structural connectivity patterns between the two types with increased connectivity in the fronto-parietal network in those with ED due to diabetes. Yang et al. compared the material properties (primary thickness, peak strain, peak stress, stiffness, viscous modulus etc. before and after continuous weight bearing) of heel pad in those with and without diabetes and investigated the impact of compressive loading history and the length of diabetes course on the material properties of heel pad. Authors demonstrated that patients with diabetes had altered material properties which may contribute to the vulnerability of heel pad to injury and ulceration.

Peng et al. presented a Case Report describing the clinical and genetic data of a Chinese patient with Werner syndrome with diabetic foot disease and myelodysplastic syndrome. They reported a novel pathogenic variation in the WRN gene in this patient with Werner syndrome.

In conclusion, this Research Topic make important contributions to our understanding of the recent advances in screening and diagnosis of DPN and may be of interest to a wider readership from the fields of Endocrinology, Neurology and General Medicine. Yet there remain important challenges in the prevention and diagnosis of DPN which future research studies could address, for e.g., novel technologies and biomarkers reported in this collection need further confirmation in large prospectively collected data, validation in external cohorts as well as in real-world settings. As suggested by Carmichael et al. such evaluation may also benefit more from fostering international collaborations rather than from the fragmented efforts of small, opportunistic studies.

## Author contributions

The author confirms being the sole contributor of this work and has approved it for publication.

## References

[1] Pop-Busui R Boulton AJ Feldman EL Bril V Freeman R Malik RA . Diabetic Neuropathy: A position statement by the american diabetes association. Diabetes Care. (2017) 40(1)136–54. 10.2337/dc16-2042 PMC6977405 27999003

[2] Selvarajah D Kar D Khunti K Davies MJ Scott AR Walker J . Diabetic peripheral neuropathy: advances in diagnosis and strategies for screening and early intervention. Lancet Diabetes Endocrinol. (2019) 7(12)938–48. 10.1016/S2213-8587(19)30081-6 31624024

